# Effect of Hydrogen-Enriched Solvents on the Extraction of Phytochemicals in Propolis

**DOI:** 10.1021/acsomega.3c01673

**Published:** 2023-04-07

**Authors:** Bayram Yurt

**Affiliations:** Department of Food Engineering, Faculty of Engineering and Architecture, Bingöl University, Bingöl 12000, Turkey

## Abstract

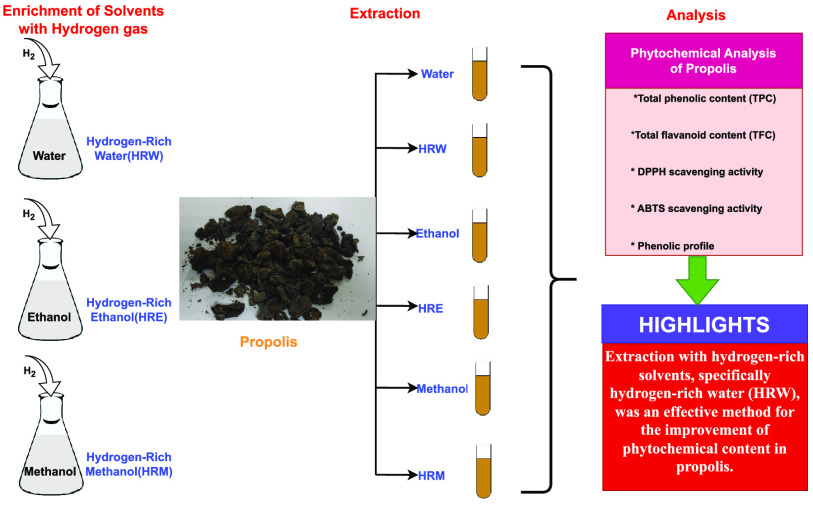

Propolis, one of the most important bee products, cannot be used in its raw form. The efficiency of the bioactive components of propolis increases with the extraction process. The choice of solvent to be used in the extraction of propolis is effective in determining the properties of the extract. Ethanol is the most widely used solvent, which significantly increases the efficiency of its bioactive components in the extraction of propolis. Effective nonalcohol-based extraction techniques have become important since alcohol-based extracts cause some discomfort and cannot be used in people with alcohol intolerance. The use of water in propolis extraction is less preferred than ethanol because it does not thoroughly dissolve the bioactive components. In this study, the effect of incorporating hydrogen into solvents (water, ethanol, and methanol) on the extraction of total phenolic content, total flavonoid content, antioxidant activities, and phenolic compound profile of the propolis sample was evaluated. Incorporation of H_2_ into water, ethanol, and methanol led to an increase in total phenolic content by 19.08, 5.43, and 12.71% and in the total flavonoid content by 28.97, 17.13, and 2.06%, respectively. Besides, the highest increases in 2,2-diphenyl-1-picrylhydrazyl (DPPH) and 2,2′-azino-bis(3-ethylbenzothiazoline-6-sulfonic acid (ABTS) scavenging activities were observed in hydrogen-rich water (4.4%) and hydrogen-rich ethanol (32.4%) compared to their counterparts, respectively. On the other hand, incorporation of H_2_ into different solvents led to significant increases in different phenolics, and it was observed that the level of change was dependent on the type of the phenolic compound and the solvent used. This study is important in terms of using hydrogen-enriched solvents to extract phenolics from propolis for the first time. Using hydrogen-rich solvents, specifically hydrogen-rich water, was observed to be an effective method for the improvement of phytochemical extraction efficiency in propolis.

## 1 Introduction

Propolis, one of the important bee products, is a resin-like product produced by bees by processing the substances they collect from coniferous trees, buds, leaf stems, and secretions of plants with the enzymes they secrete from the glands in their heads.^1,2^ Bees apply propolis on the internal walls of their hive or other cavities. It is used to block holes and cracks, to repair combs, to strengthen the thin borders of the comb, and to reduce the hive entrance in the fall or make it easier to defend. In addition, propolis is used as an embalming substance to cover hive invaders that bees kill but cannot transport out of the hive.^3,4^

Propolis has a high disinfection effect, and bees use it to disinfect the hive and comb cells. The action against microorganisms is an essential characteristic of propolis. Therefore, propolis has been used by human beings since ancient times for its pharmaceutical properties.^3^ Studies have reported that propolis has antibacterial,^5^ antiviral,^6^ anti-inflammatory,^7^ anticancer,^8^ antifungal,^9^ and antitumor^10^ properties. Biochemical properties of many propolis samples from different parts of the world have been studied and evaluated. Antioxidant and antimicrobial activities and phenolic contents of them have been investigated.^11^ Propolis is used in foods, drinks, and folk medicine to improve health and prevent diseases such as inflammation, diabetes, heart disease, and cancer.^12^ In recent years, preparations made from propolis have become increasingly popular in the development of functional foods, dietary supplements, and cosmetics.^13,14^

The chemical composition of propolis varies with the source, and over 300 chemical components, including flavonoids, terpenes, and phenolic acids, have been identified in propolis.^3,15^ Propolis is sold in a range of forms, including tablets, capsules, toothpaste, mouthwash, face creams, lotions, and solutions.^16,17^ Because of these biological activities and the various applications of propolis, the interest in elucidating the composition and biological activities increases day by day.^18^ Its chemical composition and botanical sources depend on geographical location, bee species, foraging distance, vegetation in the area, and the climatic characteristics of the site.^19^

Molecular hydrogen (H_2_) is the simplest and smallest molecule in nature. Despite its low solubility in water and fats, recent research revealed the beneficial impacts of hydrogen on the protection of antioxidants, phenolics, flavonoids, and pigments of different food products such as orange juice,^20^ polyunsaturated fatty-acid-enriched dairy beverage,^21^ acid-skimmed milk gels,^22^ fat-free fermented milk,^23^ nonfat yogurt,^24^ dried apple,^25^ dried apricot,^26^ white cheese,^27^ strawberries,^28^ and butter.^29,30^ The application of H_2_ has prevented the production of biogenic amines.^31,32^ These beneficial effects of hydrogen on the preservation of nutritional and quality charcteristics in food products were linked to hydrogen’s reducing property, small size, and high diffusion in the environment and various tissue types.^33,34^ Because of its small size and lipophilicity, hydrogen gas can passively diffuse through the cell membrane, allowing it to easily reach all the organelles within the cell.^35^ A recent study reported that the extraction of phenolic compounds from green tea leaves was found to be greater when bubbling water with hydrogen gas compared to other gases such as N_2_, CO_2_, O_2_, and air.^36^

There are no studies dealing with the impact of hydrogen-enriched solvents on the antioxidant activity, total flavonoid phenolic contents, as well as phenolic profiles of propolis extracts. Considering the lack of information in the literature, this study aimed to investigate the effect of hydrogen-enriched solvents on some phytochemicals of propolis extract.

## 2 Materials and Methods

### 2.1 Materials

The propolis samples were obtained from the Bingöl region in Turkey. The propolis samples were freeze-dried (Germany) and then ground into powder by a mortar and kept at −80 °C until analysis.

### 2.2 Chemicals

All chemicals and standards were purchased from Sigma (MO, USA) and Merck (Darmstadt, Germany).

### 2.3 Extraction of Propolis Samples

An amount of 1 g of propolis powder was added to 20 mL of solvent (ultrapure water, ethanol, methanol, hydrogen-rich water (HRW), hydrogen-rich ethanol (HRE), and hydrogen-rich methanol (HRM)) followed by a 24 h mixing phase in a shaking incubator (HZQ-X300, China) at 120 rpm and 35 °C. Hydrogen-rich solvents were prepared by bubbling pure hydrogen gas (99.99%) (from a commercial gas tank: Elite Gaz Teknolojileri, Ankara, Turkey) into the solvents at 1 L/min for 3 min. The mixtures were then filtered through filter paper, and the solvents were separated from the filtrate using a rotary evaporator (Heldolph Hei- VAP, Germany) at 35 °C for alcoholic solvents and a vacuum oven (35 °C) for aqueous ones until dry. The extracts were kept at −80 °C until analysis. All extracts were dissolved in ultrapure water at 1 mg/mL for total phenolics, flavonoids, and antioxidant activity analyses, while they were dissolved in methanol (80%) for HPLC analysis.

### 2.4 Determination of Total Phenolic Content (TPC)

The TPC analysis was carried out in accordance with López et al.^37^, with few modifications. An amount of 1 mL of extract solution was added to a mixture of 2.3 mL of ultrapure water and 1 mL of Folin- Ciocalteau’s reagent followed by a vortex step for 30 s. The mixture was then incubated in the dark at 25 °C for 5 min. Afterward, 2 mL of 7% Na_2_CO_3_ was added, and the mixture was incubated again in the dark at 25 °C for 30 min. The absorbance was read using a spectrophotometer (AQUAMATE UV–vis, China) at 765 nm. Gallic acid was used as a standard, and the TPC was calculated as gallic acid equivalent (GAE) per gram of extract.

### 2.5 Determination of Total Flavonoid Content (TFC)

The TFC was determined according to Vital et al.^38^ with minor modifications. An amount of 300 μL of extract solution was added to a mixture of 150 μL of aluminum chloride (AlCl_3_) solution (50 g/L) and 2550 μL of methanol. The mixture was kept in the dark at 25 °C for 30 min. The absorbance was then read at 425 nm. Quercetin was used as a standard, and flavonoid content was calculated as quercetin equivalent (QE) per gram of dry extract.

### 2.6 DPPH Radical Scavenging Activity Assay

The analysis of DPPH radical scavenging activity was carried out following the procedure described below: 0.5 mL of the extract solution was combined with 2.5 mL of DPPH solution (6 × 10^−5^ M) and the resulting mixture was incubated at room temperature for 90 min. The absorbance was measured at 515 nm,^39^ and the antioxidant capacity value was determined as milligrams of ascorbic acid equivalent per gram of dry extract (mg AAE/g DE).

### 2.7 ABTS Radical Scavenging Activity Assay

ABTS radical scavenging activity analysis was carried out as described below. A 7 mM ABTS solution containing 2.45 mM K_2_S_2_O_8_ was prepared and kept in the dark at room temperature for 12–16 h. A 20 mM sodium acetate solution was prepared, and its pH value was adjusted to 4.5 with 0.1 N HCl. The prepared ABTS solution was then diluted with 20 mM sodium acetate to obtain an absorbance of 0.700 ± 0.01 at 734 nm. An amount of 100 μL of extract solution was mixed with 1900 μL of ABTS solution, and at the end of 5 min, the absorbance was measured at 734 nm.^40^ Trolox was used as a standard, and the ABTS radical scavenging activity was calculated as mg of Trolox equivalent (TE) per g of dry extract (mg TE/g DE).

### 2.8 Phenolic Profile Analysis

The phenolic profile of the propolis extracts was performed using RP-HPLC according to the method described by Kocabey et al.^41^ with few modifications. An amount of 20 μL of extract samples was injected into the column (ODS3, 250 × 4.6 mm, 5 μm; GL Science, Tokyo, Japan) set at 28 °C with a flow rate of 1 mL/min. Two mobile phases were used with solvent A (0.1% H_3_PO_4_ in water) and solvent B (0.1% H_3_PO_4_ in acetonitrile). The flow rate of the mobile phase started at 8% (solvent B) and increased to 11% within 4 min. Subsequently, the flow rate of solvent B was adjusted to 35% at 25 min, 60% at 30 min, and 35% at 45 min and returned to 11% at 50 min and 8% at 55 min. Detection was carried out at wavelengths of 280, 320, and 360 nm. Authentic phenolic standards were used for the calculation of each individual phenolic content.

### 2.9 Statistical Analysis

Results were subjected to one-way ANOVA with GraphPad Prism 9 Software, and Tukey’s posthoc test was applied using the IBM SPSS Statistics 26 package program. Statistically significant differences were considered at the level of *p* < 0.05. The experiments were performed in duplicate, and the analyses were performed in triplicate (*n* = 3).

## 3 Results and Discussion

### 3.1 Total Phenolic Content (TPC)

The TPC values of propolis extract obtained by pure solvents (water, ethanol, methanol) and hydrogen-rich solvents (HRW, HRE, HRM) are shown in Table 1.

**Table 1:** Total Phenolic and Flavonoid Contents and Antioxidant Capacity of Propolis Extracts Obtained by Different Solvents

Solvent	TPC (mg GAE/g extract)	TFC (mg QE/g extract)	DPPH scavenging activity (mg AAE/g extract)	ABTS scavenging activity (mg TE/g extract)
Water	193.51 ± 45.71^a^	68.70 ± 3.21^a^	86.04 ± 2.91^a^	690.03 ± 1.10^a^
HRW	230.43 ± 31.06^a^	88.60 ± 11.56^a^	89.83 ± 1.59^a^	692.03 ± 0.04^a^
Ethanol	127.47 ± 0.64^a^	285.18 ± 37.49^a^	92.47 ± 0.03^a^	450.37 ± 28.97^a^
HRE	134.39 ± 19.05^a^	334.04 ± 3.93^a^	93.09 ± 0.09^a^	596.16 ± 37.95^a^
Methanol	150.80 ± 1.82^a^	368.45 ± 11.75^a^	91.50 ± 0.09^a^	585.18 ± 12.98^a^
HRM	169.96 ± 3.75^a^	376.03 ± 12.01^a^	92.38 ± 0.09^a^	624.12 ± 5.99^a^

a For each solvent type in the same column, different lowercase letters indicate a significant difference between the solvents (*p* < 0.05). HRW, hydrogen-rich water; HRE, hydrogen-rich ethanol; HRM, hydrogen-rich methanol.

The highest level of the TPC of propolis was found for water in the nonhydrogenated solvent group and for HRW in the hydrogen-rich solvent group with 193.51 and 230.43 mg of GAE/g of extract, respectively (*p* > 0.05). The lowest TPC was observed for ethanol and HRE within the hydrogen-rich solvents with the values of 127.47 and 134.39 mg of GAE/g of extract, respectively (*p* > 0.05). However, the TPC level of hydrogen-rich methanol (169.96 mg of GAE/g of extract) was found to be higher than that of methanol (150.80 mg of GAE/g of extract) (*p* < 0.05).

When H_2_ was infused into solvents, the TPC of propolis extract was increased by 19.08%, 5.43%, and 12.71% for water, ethanol, and methanol, respectively (Table 1). Thus, the incorporation of H_2_ into solvent led to the highest increase in the TPC shown for HRW followed by HRM and HRE. These results emphasize that hydrogen incorporation into methanol could be a good alternative for extracting phenolics from propolis samples. H_2_ can be dissolved in water with an oxidoreduction potential value (*E*_h_) of −283 mV, giving a reducing property that can help to preserve redox homeostasis inside the cell, allowing it to protect phenolics and antioxidants from oxidative reactions.^40^

### 3.2 Total Flavonoid Content (TFC)

In Table 1, the TFC of propolis extract obtained by pure solvents (water, ethanol, and methanol) and hydrogen-rich solvents (HRW, HRE, and HRM) is shown. The highest level of the TFC of propolis extract was found for methanol in the nonhydrogenated solvent group and for HRM in the hydrogen-rich solvent group with 368.45 and 376.03 mg of QE/g of extract, respectively (*p* > 0.05). On the other hand, the lowest TFC was observed for water and as 68.70 and 88.60 mg of QE/g of extract, respectively (*p* < 0.05). When H_2_ was infused into solvents, an increase in the TFC of propolis extract by 28.97%, 17.13%, and 2.06% for water, ethanol, and methanol, respectively, could be observed (Table 1). These results emphasize that the effect of hydrogen incorporation into water is the most potent method for extracting flavonoids from the propolis sample. Similar results have been reported in extracts of sour cherry puree where the highest levels of TFC were established in pure methanol extracts.^41^

### 3.3 DPPH Scavenging Activity

The DPPH (2,2-diphenyl-1-picrylhydrazyl) radical scavenging activity of propolis extract obtained by different solvents (water, ethanol, and methanol) and hydrogen-rich solvents (HRW, HRE, and HRM) are given in Table 1. The highest level of DPPH scavenging activity of propolis extract was obtained for ethanol in the non-hydrogenated solvent group and HRE in the hydrogen-rich solvent group with 92.47 and 93.09 mg of AAE/g of extract, respectively (*p* < 0.05). However, the lowest DPPH scavenging activity was observed for water and HRW with the values of 86.04 and 89.83 mg AAE/g extract, respectively (*p* > 0.05). The incorporation of hydrogen into the solvent led to the highest increase in DPPH scavenging activity observed for HRW followed by HRM and HRE. Incorporation of H_2_ into water, ethanol, and methanol led to an increase in TPC by 4.40, 0.96, and 0.67%, respectively. These results reveal that the effect of hydrogen incorporation into ethanol and methanol could be a good option when the extraction of antioxidants is targeted. These results show that the reducing properties of H_2_ might protect the antioxidants that are sensible to oxidation reactions during the extraction process.

### 3.4 ABTS Radical Scavenging Activity

The ABTS (2,2′-azino-bis(3-ethylbenzothiazoline-6-sulfonic acid) radical scavenging activity of propolis extracts obtained by different solvents (water, ethanol, methanol) and hydrogen-rich solvents (HRW, HRE, HRM) are shown in Table 1. The highest level of ABTS scavenging activity of propolis extract was found for water in the nonhydrogenated solvent group and HRW in the hydrogen-rich solvent group with 690.03 and 692.03 mg of TE/g of extract, respectively (*p* > 0.05). However, the lowest ABTS scavenging activity value was observed in ethanol and HRE extracts with the values 450.37 and 596.16 mg of TE/g of extract, respectively (*p* < 0.05) (Table 1). The incorporation of H_2_ into solvents led to an increase in the ABTS radical scavenging activity of propolis extract by 32.37% for HRE and 6.65% for HRM, respectively (*p* < 0.05) (Table 1). These results reveal that H_2_ incorporation into ethanol is the most potent method for improvement of antioxidant extraction in propolis as obtained by the ABTS radical scavenging activity method.

### 3.5 Phenolic Profile Analysis

Phenolic profiles of propolis extracts are shown in Table 2. Regarding nonflavonoids and hydroxycinnamic acids, gallic acid was better extracted by HRW followed by water. The gallic acid contents of pure water solvent and HRW in the hydrogen-rich solvents were observed as 36.73 and 43.53 mg/L, respectively (*p* < 0.05). The incorporation of H_2_ into solvents led to a significant increase in the gallic acid content of propolis extract by 18.51% for HRW. This result reveals that H_2_ incorporation into water is the most potent method for gallic acid content of propolis extract. On the other hand, the chlorogenic acid contents of water and HRW extracts were found to be 4.17 and 9.21 mg/L, respectively (*p* < 0.05). The incorporation of H_2_ into solvents led to a significant increase in the chlorogenic acid content of propolis extract by 120.86% for HRW. Regarding flavonoids and flavan-3-ols, catechin contents of pure water solvent and HRW in the hydrogen-rich solvents were observed as 13.70 and 21.12 mg/L, respectively (*p* < 0.05), corresponding to an increase of 54.16% being the highest in hydrogen-rich water. On the other hand, epicatechin contents of pure water solvent and HRW in the hydrogen-rich solvents were observed as 75.58 and 104.36 mg/L, respectively (*p* < 0.05). The incorporation of H_2_ into solvents led to a significant increase in the gallic acid content of propolis extract by 38.10% for HRW.

**Table 2:** Phenolic Profile Analysis (μg/g of Extract) of Propolis Extract Prepared by Pure and Hydrogen-Rich Solvents

Solvents	Gallic Acid	Chlorogenic Acid	Catechin	Epicatechin	Caffeic acid	Rutin	*p*-Coumaric Acid	*trans*-Ferulic Acid	Rosmarinic Acid	Quercetin
Water	36.73 ± 2.69^a^	4.17 ± 0.41^a^	35.35 ± 5.69^a^	75.58 ± 5.01^a^	113.01 ± 12.95^a^	2.37 ± 0.52^a^	11.08 ± 0.42^a^	65.01 ± 0.46^a^	0.04 ± 0.01^a^	1.59 ± 0.24^a^
HRW	43.53 ± 4.99^a^	9.21 ± 0.23^a^	55.30 ± 11.57^a^	104.36 ± 11.22^a^	420.87 ± 59.72^a^	2.27 ± 0.08^a^	49.74 ± 8.99^a^	112.09 ± 8.04^a^	10.36 ± 1.74^a^	1.72 ± 0.18^a^
Ethanol	1.28 ± 0.11^a^	n.d.	15.31 ± 2.22^a^	3.56 ± 0.33^a^	25.64 ± 1.83^a^	10.28 ± 1.67^a^	6.41 ± 0.71^a^	20.15 ± 3.10^a^	0.04 ± 0.01^a^	15.11 ± 1.22^a^
HRE	4.35 ± 0.85^a^	n.d.	15.03 ± 1.62	3.28 ± 0.18^a^	30.79 ± 3.16^a^	12.00 ± 0.21^a^	07.39 ± 0.62^a^	22.22 ± 2.28^a^	1.05 ± 0.25^a^	16.90 ± 1.37^a^
Methanol	4.89 ± 0.45^a^	n.d.	13.70 ± 0.63^a^	0.31 ± 0.07^a^	23.16 ± 3.93^a^	13.22 ± 0.87^a^	05.67 ± 0.44^a^	19.46 ± 2.90^a^	1.15 ± 0.31^a^	15.50 ± 1.15^a^
HRM	5.73 ± 0.37^a^	n.d.	21.12 ± 1.74^a^	5.28 ± 1.19^a^	40.03 ± 3.53^a^	15.28 ± 1.70^a^	10.02 ± 1.16^a^	31.95 ± 1.04^a^	1.26 ± 0.18^a^	21.02 ± 2.18^a^

a For each solvent type in the same column, different lowercase letters indicate a significant difference between the solvents (*p* < 0.05). HRW, hydrogen-rich water; HRE, hydrogen-rich ethanol; HRM, hydrogen-rich methanol.

Regarding nonflavonoids and hydroxycinnamic acids, caffeic acid–acid was better extracted by HRW followed by water. The caffeic acid contents of pure water solvent and HRW in the hydrogen-rich solvents were observed as 113.01 and 420.87 mg/L, respectively (*p* < 0.05). The incorporation of H_2_ into solvents led to a significant increase in the caffeic acid content of propolis extract by 272.42% for HRW. This result reveals that H_2_ incorporation into water is the most potent method for caffeic acid content of propolis extract. On the other hand, for rutin content of pure methanol solvent, HRM in the hydrogen-rich solvents was observed as 13.22 and 15.28 mg/L, respectively (*p* < 0.05). The incorporation of H_2_ into solvents led to a significant increase in the rutin content of propolis extract by 15.58% for HRM.

*p*-Coumaric acid contents in water solvent and HRW extracts were found to be 11.08 and 49.74 mg/L, respectively (*p* < 0.05). The incorporation of H_2_ into solvents led to a significant increase in the *p*-coumaric acid content of propolis extract by 321.53% for HRW. This result reveals that H_2_ incorporation into water is the most potent method for *p*-coumaric acid content of propolis extract. *trans*-Ferulic acid content was found to be 65.01 and 112.09 mg/L in water and HRW extracts, respectively (*p* < 0.05). The incorporation of H_2_ into solvents led to a significant increase in the *trans*-ferulic acid content of propolis extract by 72.18% for HRW.

Another phenolic acid, rosmarinic acid, was observed to be at low concentrations in water and ethanolic extracts (0.04 mg/L), but when water is hydrogenated the values significantly increase and reach values of 10.36 mg/L. The incorporation of H_2_ into water led to a significant increase in the *trans*-ferulic acid content of propolis extract by 25800% for HRW. Quercetin, regarding flavonoids and Flavonoid glycosides, was observed for pure methanol solvent and HRM in the hydrogen-rich solvents with 15.50 and 21.02 mg/L, respectively (*P* < 0.05). The incorporation of H_2_ into solvents led to a significant increase in the quercetin content of propolis extract by 35.61% for HRM. This result reveals that H_2_ incorporation into methanol is the most potent method for quercetin content of propolis extract.

More than 600 natural components have been identified in propolis, and due to this rich composition it is accepted as biologically active and healthy. The importance of propolis has increased even more after the SARS CoV-2 epidemic in the world. However, propolis cannot be used in its raw form in the industry due to its complex structure, sticky, resinous nature, and strong taste and smell. Therefore, propolis extraction is the primary and most critical process required for its consumption. The use of many solvents has been reported in the literature for the extraction of antioxidants and phenolic compounds in propolis, ethanol being the most commonly used one due to its greater dipole moment.^42−44^ Besides, several other solvents including water,^45,46^ methanol,^47,48^ methylene chloride,^47^ dichloromethane,^49^ hexane,^50^ ethyl acetate,^51^ acetone,^46^ olive oil,^52^ β-cyclodextrin,^52^ dimethylsulfoxide,^46,53^ and chloroform^54−57^ have also been reported. Regarding the effect of solvents, there are different results in the literature which may be due to the extraction method used and the properties of the extracted propolis, leading to differences in the total phenolic and flavonoid contents, antioxidant capacity, and phenolic profile. The efficiency of the phenolic compounds and antioxidant activity of propolis increases with the extraction process.^55^ Therefore, the solvent is accepted as one of the most important parameters.

Molecular hydrogen is a colorless, odorless, tasteless, flammable, and nontoxic gas. Molecular hydrogen is dissolved directly in water, ethanol, and methanol to be used in the form of hydrogen-rich water (HRW), hydrogen-rich ethanol (HRE), and hydrogen-rich methanol (HRM). This study focused on the effect of hydrogen-enriched solvents on the antioxidant activity, total flavonoid and phenolic content, as well as phenolic profile of propolis. The effect of hydrogen incorporation into methanol (HRM) was observed to be the most potent method for extracting the total phenolics from the propolis sample. On the other hand, incorporation of H_2_ into water (HRW) resulted in the highest results in terms of total flavonoids indicating that the extraction efficiency of phenolic acids and flavonoids might be different in different solvents. In the case of antioxidant activity, hydrogen incorporation into ethanol (HRE) and methanol (HRM) provided better results.

Another important finding of this work is that depending on the type of the phenolic the highest values were obtained in different solvent systems, showing the selectivity of phenolic compounds. For example, HRW was the best extraction solvent for gallic acid, chlorogenic acid, caffeic acid, *p*-coumaric acid, *trans*-ferulic acid, rosmarinic acid, catechin, and epicatechin, whereas HRM yielded the best result for quercetin in propolis samples. Considering these findings, it should be noted that the solvent selection should be made, taking the phenolic profile of the samples into account to be able to obtain the best results.

The exact mechanism of action by which H_2_ improves the extraction capacity of bioactive compounds in propolis is not clear, but it could be partly due to its reducing capacity, solubility in hydrophobic phases, and easiness of diffusion through tissues. Thus, it is also not clear if the increase in phytochemicals observed in samples extracted with hydrogen-rich solvents in the present study was due to the protection of phenolic compounds from enzymatic and nonenzymatic oxidative reactions or by the liberation of cell-wall-bound phenolic substances or maybe by a combination of both processes.

## 4 Conclusion

In this study, hydrogen-rich solvent systems were used and compared with their counterparts. According to the results, hydrogen-rich solvents were observed to be effective in terms of improving the phenolic content and antioxidant activity. Hydrogen-rich water (HRW) extraction was efficient specifically for the improvement of total phenolics and antioxidant activity measured by the ABTS method, whereas hydrogen-rich ethanol (HRE) and hydrogen-rich methanol (HRM) showed higher results for total flavonoids and antioxidant activity measured by the DPPH method. In further studies, more extensive research on the effect of hydrogen-enriched solvents for the improvement of the phytochemical extraction efficiency should be performed. Indeed, safety issues should also be considered for widening its application and especially use in the food and nutraceutical industries.
